# Segmenting Young Adult University Student’s Eating Behaviour: A Theory-Informed Approach

**DOI:** 10.3390/nu11112793

**Published:** 2019-11-15

**Authors:** Anna Kitunen, Sharyn Rundle-Thiele, Julia Carins

**Affiliations:** 1 Social Marketing @ Griffith, Griffith University, 170 Kessels Road, Nathan, QLD 4111, Australia; s.rundle-thiele@griffith.edu.au (S.R.-T.); j.carins@griffith.edu.au (J.C.); 2 Food and Nutrition, Land Division, Defence Science and Technology, Scottsdale, TAS 7260, Australia

**Keywords:** social marketing, young adults, behaviour change, segmentation, eating behaviour

## Abstract

The purpose of this paper is to extend behavioural theory and segmentation application. Specifically, this paper draws on three segmentation bases and behavioural theory that extends focus beyond individual psychological predispositions to form segments within the healthy eating context for young adult university students (20–35 years) in Queensland, Australia. Participants were invited to take part in an online survey via email and through face to face intercept to ensure a diverse cross section was obtained. Structural equation modelling revealed that the Motivation, Opportunity, and Ability (MOA) framework can be utilised to explain healthful eating behaviour and two-step cluster analysis uncovered two distinct segments with education, motivation to eat healthily and Turconi’s eating behaviour scores being the most important variables within the wider multivariate segment formation. This paper contributes to literature in the following ways. First, it confirms the importance of behavioural bases in segment formation and supports inclusion of other bases, namely demographics and psychographics. Next, it provides evidence of the value of including behavioural theory, which extends focus beyond what individuals think to understand how the environment may support them. Finally, this paper demonstrates that the MOA framework together with eating behaviour and demographic factors (education) can produce theoretically informed segments.

## 1. Introduction

Obesity is a growing issue both in Australia and globally, generating significant health and economic consequences costing individuals and community [1]. Young adulthood (early 20s to 30s) has been identified as a key stage in life where individuals are at greatest risk of weight gain [2,3]. Overweight and obesity results in considerable decreases in life expectancy and increases in early mortality among adults. Overweight and obesity are described by an excess of body fat and are usually diagnosed according to a body mass index (BMI) equal to or greater than 25 kg/m^2^ (overweight) and greater than 30 kg/m^2^ (obesity). Early onset obesity has been estimated to reduce life expectancy by seven years at the age of 40 [4]. A recent study found that among 25-year-old Australians, a 4.2- and 3.6-year decrease in life expectancy was observed in overweight men and women while the numbers were even higher for obese men and women (8.3 and 6.1 years) [5]. Overweight and obesity occurs when an individual’s calorie intake exceeds calories expended on a serial basis over time. However, it is commonly acknowledged that obesity is a complex multi-factorial issue with affected individuals influenced by a multitude of societal and biological factors including food production, societal influences, food consumption, individual psychology, biology, individual activity, and the surrounding environment [6].

Given obesity is a chronic condition that builds over a sustained period of time, one-off treatments cannot be expected to counteract or reverse this condition. The complex, multifactorial nature of obesity requires sustained efforts at all levels to deliver change [7], with prevention efforts forming a critical element. For example, Garcia-Moll [8] identified that once an individual becomes obese, the prognosis of them returning to, and maintaining a healthy weight is poor. The Garcia-Moll [8] study suggests that 2–5% of obese individuals will successfully keep off 10% of their body weight without medical intervention. A review of the literature indicates that education [9,10], health [11,12] and policy [13] initiatives dominate obesity prevention. The literature indicates that a key failure of many overweight and obesity prevention programs is a focus on desired health outcomes [14,15]. Health focused messaging and programs fail to engage and retain young adults because these programs are perceived to be irrelevant [16], which in turn further limits ability to deliver sustained weight loss effects in this age group [17]. Given society cannot afford expensive health care models, alternative, cost-effective delivery models are needed to supplement current efforts; and there is a specific need for programs that engage young adults.

In order to develop effective and engaging programs for young adults, it is important to understand the many and varied barriers and enablers of healthful eating for young adults; and then design behaviour change programs tailored to their needs [18]. However, dominant behaviour change practice overlooks this complexity implementing approaches that target general populations utilising a one-size-fits-all approach [19]. A one-size-fits-all approach limits program effectiveness, given large numbers of the audience may be left dissatisfied, uninterested, or unchallenged [20]. A recent study focusing on young adults found that a one size fits all approach failed to improve eating habits [21]. Moreover, research indicates that different segments respond to programs differently [22,23]. Segmentation is an analytical approach commonly used within commercial and social marketing to determine if sub-groups exist within a larger group. The approach uses segmentation ‘bases’ (four key areas—demographics, psychographics, behaviour, and geography) to categorize individuals into groups that are mostly similar within the group, but different across groups (see Dietrich et al. [24] for a detailed resource on segmentation).

Within segmentation in social marketing, theory is rarely used, and when theory has been applied studies mainly use theories that focus on how individuals think [25,26,27]. A review of the literature indicates that theoretically designed programs may deliver stronger results [28]. Behavioural theories can improve the value of market segmentation by assisting to identify factors that influence individual behaviour for different segments [29]. This proposal is consistent with evidence indicating that programs that promote the refusal of risky behaviours and the adoption of protective behaviours are more likely to be effective if they are theoretically informed [30]. Therefore, the current research aims to extend existing literature by applying a behavioural theory in the segmentation process to offer additional insights for improving the eating habits of young adults. The Motivation, Opportunity, and Ability (MOA) framework [31] was chosen as it permits application of a wider socio-ecological perspective, which is needed to capture the complexity experienced by young adults for eating, given that environments surrounding people and levels of social support differ [32].

Taken together, delivery of programs to encourage healthful eating targeted to group differences and accommodating a wider socio-ecological perspective are needed to more effectively engage with a wider cross section of young adults. To address these gaps in the literature, the aims of this study are threefold. First, this study aims to examine which segmentation bases have previously been used by social marketers to derive segments. Second, this study identifies theories that have previously been used to inform segment solutions. Finally, drawing on the findings under the first two aims, and the need to extend research focus beyond individuals targeted for change to ensure that consideration is given to environmental support for healthful eating, this paper applies the MOA framework to understand if segments are evident in a young adult population.

### Segmentation and Social Marketing

Similar to commercial marketing, social marketers aiming to change behaviour for social good can find groups of individuals most in need [33], or most able or willing to change their behaviour through segmentation [29]. What remains to be tested is whether effectiveness and efficiency of social marketing efforts can be increased by applying segmentation [34,35,36]. This is especially relevant given social marketing programs are often supported with limited human and financial resources [37]. Despite this, only a few social marketing studies have applied segmentation [23,36,38,39]. These studies indicate that distinct segments do exist within social contexts and that when evaluated at a group level, segments do respond differently to social marketing programs [22,23].

Several approaches can be used to segment target populations to form smaller groups with similar wants, needs, or behaviours. However, each approach uses a number of ‘bases’ to determine how separation into smaller groups can be achieved. The components that can be utilised in segmentation analysis can be classed into four main bases, namely demographic, geographic, psychographic, and behavioural [40]. The underlying assumption of segmentation studies is that populations differ from one another, exhibiting different individual characteristics, attitudes, motivations, lifestyles, places lived, and behaviours [41]. Table 1 summarises the self-identified social marketing studies encouraging healthful eating that reported using segmentation prior to program implementation.

Table 1 shows that all of these healthful eating studies used only one or two segmentation bases —mainly demographic and psychographic. In social marketing, demographic and geographic are the oldest and most frequently used bases across all behavioural contexts [53], and only recently has the focus shifted more towards psychographic and behavioural variables [22,23,54,55,56]. Recent research indicates that psychographic and behavioural variables are most important in cluster formation based on the predictor importance score while demographic variables were less important [22]. This finding is supported in a study focusing on active school travel stating that both psychographics and behavioural variables were significant in cluster formation and that four variables representing psychographics, behavioural, and geographic bases were highly important in forming the segments in a walk to school context among caregivers [39].

Only a few studies have used segmentation in social marketing aiming to encourage healthful eating as it relates to targeting overweight an obesity [44,45,54]. Differences in attitudes towards healthful eating among 13–15-year-old adolescents were found using different segmentation models [45]. Chrysochou et al. [44] used demographics and psychographics to identify three segments of adults based on their attitudes towards healthful eating. The attitudinal beliefs of these three segments offered insights for tailoring social marketing programs to encourage healthful eating. Van Loo et al. [51] investigated psychographic variables to identify four segments in four European Union countries (UK, Germany, Belgium, and the Netherlands) based on their involvement to eat healthily and sustainably. Each study emphasizes the importance of a segmented social marketing approach to encourage healthful eating.

The limited investigation and application of segmentation, especially as it relates to improving the health of young adults, emphasises the need for further research in this area. Furthermore, the segmentation process may be enhanced by the incorporation of behavioural theory which could assist in the identification of factors that influence behaviour change in different segments [29]. The application of theory during segmentation is discussed next.

## 2. Theoretical Framework

Reviews have shown that theory use in social marketing is limited with only 23% of interventions being guided by theory [26,27]. When theory is used in social marketing studies, individual-focused theories such as the Theory of Planned Behaviour and the Health Belief Model dominate [25]. Only 21% of interventions using theory sought to investigate the broad contextual and environmental factors that have an impact on the target audience’s behaviour, and these were mainly based on Social-Cognitive Theory or the Ecological Model [27]. Eating behaviour is complex and individuals are subjected to social and environmental influences, suggesting individual-focussed and rational decision models may limit insights to inform behaviour change planning. A recent review identified that behaviour change techniques that change the environment demonstrated the potential of positively changing eating behaviour [57]. Taken together, this indicates integration of theories that recognise the complexity of eating behaviour, along with application of segmentation, have the potential to enhance social marketing practice.

Few social marketing segmentation studies encouraging healthful eating (see Table 1) report theory use. Moreover, when theory use is reported a failure to explicitly report how theory was used is evident. The importance of incorporating behavioural theories in market segmentation is highlighted in a physical activity context [39]. The Theory of Planned Behaviour was used to distinguish segments in a study aiming to change children’s walking behaviour [39]. This finding is further supported in a study where Theory of Interpersonal Behaviour was used to segment the water use market [38].

The MOA framework has been previously used within social marketing in a land management context where it was suggested that the MOA framework could provide a basis for segmentation [31]. Previously, the MOA framework has been applied to examine the interrelated effect of motivation, opportunity, and ability on the behavioural outcomes in weight management context (nutrition and physical activity) among Australian adults [58]. The results revealed that 63% of the participants who reported learning new habits or adopted changes to their mindset, reported weight loss following program participation. Findings also showed an important relationship between new habits formed and changes adopted from participation in the weight management program and participant’s health and wellbeing outcomes including weight loss following participation in the program [58]. This suggests that the MOA framework is suitable for improving understanding of the market for changing eating habits.

The MOA framework has been used to classify consumers into distinct categories based on how prone, resistant, or unable the consumer is to change depending on their motivation, opportunity, and ability to perform the target behaviour [31]. Motivation consists of the internal drivers to perform a behaviour. Motivation incorporates willingness, readiness, desire, and interest to engage in information processing [59] or a particular behaviour [60]. It has been proposed that goals and needs, as well as, perceived risk and alignment with existing attitudes impact motivation [61]. Ability refers to the extent which people possess the required capabilities or skills to engage in a particular behaviour to achieve the outcome [60,61]. Self-efficacy [62] is a determinant of ability [31] and includes individual’s beliefs about their capabilities to organize and perform a series of actions [62]. Self-efficacy is likely to be affected by environmental influences such as cultural norms and it has been linked to motivation to perform a behaviour [63]. Opportunity refers to the extent to which external factors mitigate or prevent engaging in a specific behaviour [60]. Consequently, lack of opportunity includes situations where an individual can be motivated to act but is prevented from doing so by environmental factors [31], e.g., low or no supply of healthy alternatives.

In summary, there is a need for research that investigates the application of segmentation to inform the design of programs to increase healthful eating by young adults; and there is potential for behavioural theory that extends focus beyond individual psychological predispositions to enhance the segmentation process. Therefore, this study adopts an approach that uses three segmentation bases—namely, demographic, psychographic, and behavioural—while simultaneously incorporating the MOA theoretical framework.

## 3. Materials and Methods

### 3.1. Target Population

The sample of young adults aged 20–35 was sourced from universities in South East Queensland, Australia. Participation in the survey was voluntary and completely anonymous, and the survey took approximately 15 min to complete. Participants were invited to complete the survey face to face on an iPad on university market days on campus in August 2017. Online advertising was also used to invite further participants from multiple universities in September 2017 to ensure maximum heterogeneity in the university-based young adult sample. Following the recommendations of Dillman et al. [64], respondents were offered an equal chance of winning one of five AUS$50 gift vouchers to incentivise their participation. Ethical approval was obtained for the research on 4 July 2017 from the Human Research Ethics Committee (approval no. 2017/504) at Griffith University.

### 3.2. Online Survey

The first section of the survey assessed food habits and eating behaviour related specifically to breakfast intake, number of meals a day, daily consumption of fruit and vegetables, and daily consumption of both desserts and alcohol [65]. Eating behaviour was measured with eight, four-point Likert scale items with the following categories: never, sometimes, often and always. Each of the answer options represented a score from zero to three with the highest score assigned to the healthiest option and lowest score to the least healthy option. Following the method used in Turconi et al. [65] an overall eating behaviour score was generated (0 being the lowest and 24 being the highest possible score) for each participant. The second section of the survey captured motivation, opportunity and ability to eat healthily. Motivation was measured through six previously validated items [66] for motivation (health and weight control), and newly developed items for opportunity (*n* = 3) and ability (*n* = 3) (shopping and cooking fruit and vegetables). An expert panel of 55 marketing and social marketing researchers was formed to examine the face and discriminant validity of both previously validated and newly developed items. Panel members evaluated each item and selected the MOA construct that they believed best matched each item. For the item to be kept, a minimum of 50% of experts should agree that the item is distinctly represented [67]. For this study, a cut-point of 80% agreement was set to ensure clarity that a specified item measured a specific construct. Seven-point Likert scales were used with options—never, rarely, occasionally, sometimes, frequently, usually, and always—to measure participants’ motivation, opportunity, and ability to eat healthily. In the final section of the survey, participants were asked a number of demographic questions.

### 3.3. Sample

A purposive sample of 475 young adults was collected. The sample consisted mostly of high school educated (48.6%), women (77.1%). The largest proportion of respondents was 21-year-olds (11.6%) followed by 20- and 23-year-olds (both 10.4%). There was a positive correlation between age and education (*r* = 0.56, *p* = 0.000) with 78% of high school level educated people being 20–25-year-olds and 67% of Bachelor Degree level educated people being 23–34-year-olds. Approximately half (55.4%) of the sample reported that they always eat breakfast and nearly half (43.1%) reported that they always eat at least two portions (200 g) of vegetables daily, while less than half (43.1%) reported eating at least two portions (200 g) of fruit daily indicating only sometimes.

### 3.4. Data Analysis

The study used three segmentation bases, namely demographic, psychographic and behavioural measures. Previous studies have shown that when compared to the other three base measures, geographic measures have been less important in segment formation [22,39].

First, the reliability and validity of the MOA constructs was examined. All of the three measures demonstrated high internal consistency exceeding recommended Cronbach’s alpha levels (motivation α = 0.80, opportunity α = 0.74 and ability α = 0.75). Structural equation modelling was used with AMOS Graphics version 24 to examine the measurement and structural models to understand if the MOA framework could be used to explain healthful eating behaviour. The MOA framework explained 38.3% (R^2^) of the variation in healthful eating with motivation having a significant impact on healthful eating behaviour indicating that the MOA framework offered a partial explanation of healthful eating behaviour (C.R. value > 1.96 and *p*-value < 0.01 [68,69]). Model fit indices revealed a good model fit; 1.757 (CMIN/DF), 0.969 (TLI), 0.979 (CFI), 0.064 (RMSEA), and 52.7 (Chi-square) [70,71].

After data collection and cleaning, two-step cluster analysis was performed with IBM SPSS version 25 conforming to the process designed by Norušis [72] to detect homogenous subgroups in the study population. Previously, this method has been utilised in identifying segments in health behaviours [73], psychological stress management [74], adolescent populations [23], and in physical activity behaviours [36,75]. Two-step cluster analysis is an appropriate method for this exploratory study, especially since the number and the members of the clusters are not known [76]. Furthermore, two-step cluster analysis is the only type of cluster analysis in IBM SPSS that allows simultaneous analysis of both categorical and continuous data offering an ability to simultaneously address a wide and diverse range of measures, and the capability to handle large sample sizes [72,76] which is highly relevant in the current study. The analysis was executed with five segmentation variables with low to zero correlations and the measure to respondent ratio was within the recommended guidelines [77]. Initially, log-likelihood distance was used to group the cases into pre-clusters [78]. The pre-clusters were further reduced to the best number of clusters based on the Schwartz’s Bayesian information criterion (BIC) [72]. Segments were validated in a split sample to warrant the consistency of the cluster formation in a half-sized sample. Independent sample *t*-test and Chi-square tests were executed on the categorical and continuous variables to examine cluster differences.

## 4. Results

Two distinct segments, termed ‘breakfast skippers’ and ‘weight conscious’, were uncovered with two-step cluster analysis within the sample using five variables, including three MOA variables. The analysis produced a sample (*N* = 327) with a silhouette coefficient of 0.1 that is a measure of cohesion and separation, and corresponds to other market segmentation studies [79]. Input importance levels of every segmentation variable were higher than 0.0, thus, indicating that each measure contributed to some variation within segments. Independent sample *t*-tests and chi-square tests confirmed significant differences across the five segmentation variables between the segments. The same BIC measure and number of clusters were determined when the dataset was split in half for further validation of the segment solution.

The importance of a variable to segment formation is indicated by the importance levels. Rating levels between 1.0 and 0.8 represent a highly important variable, while ratings between 0.2 and 0.0 indicates the variable was less important [72]. The highest education level completed was the most important variable in segment formation with predictor importance score of 1.0, followed by motivation (0.04), Turconi eating behaviour score (0.03), and ability and opportunity both (0.02). Each segment will now be discussed in turn drawing on wider available research data to obtain a detailed understanding of the identified segments (see Table 2).

The two segments discovered in this study were approximately equally sized with Segment 1 (breakfast skippers) (48.6%) being marginally smaller than Segment 2 (weight conscious) (51.4%). The findings indicate that Breakfast skippers consisted of high school level educated people, the majority of whom were 20–24-year-olds (77.7%). The respondents in this segment reported the lowest motivation to eat healthily (M = 4.4, SD = 1.4) and had lower perceptions of their ability (M = 4.5, SD = 1.5) and opportunity (M = 4.3, SD = 1.5) to eat healthily. In addition to feeling less motivated and perceiving they had lower ability and opportunity to eat healthily Breakfast skippers reported a lower Turconi eating behaviour score (M = 15.9, SD = 3.6) for healthful eating habits when compared to weight conscious (M = 16.9, SD = 3.1). In line with these results, breakfast skippers prefer frequently eating foods that are easy to prepare and are the most convenient option (both M = 5.1, SD = 1.1). Respondents in this segment also frequently opt for food that is inexpensive (M = 4.8, SD = 1.5), on sale (M = 4.9, SD = 1.3), and because they do not want to spend any more money (M = 4.5, SD = 1.6). They also frequently consume foods that they grew up with (M = 4.8, SD = 1.3) and sometimes chose foods because the food belongs to a certain situation (M = 3.7, SD = 1.5). Interestingly, breakfast skippers occasionally consume food because it is trendy (M = 2.8, SD = 1.4).

The second segment, weight conscious, consisted of mostly 25–29 year olds (40%) and the highest education level completed in this segment was a Bachelor’s degree (38.7%). The respondents in the weight conscious segment hold a stronger motivation to eat healthily (M = 4.9, SD = 1.2), and a stronger belief that their ability (M = 4.9, SD = 1.5) and opportunity (M = 4.7, SD = 1.5) to eat healthfully are high. Taken together, analysis of theory informed segmentation results indicate the role the eating environment (opportunity) has on eating motivations across the two segments. This segment reported sometimes consuming food because it was on sale (M = 4.4, SD = 1.3), inexpensive (M = 4.3, SD = 1.6), or because they did not want to spend any more money (M = 4.0, SD = 1.7). When compared to breakfast skippers, the weight conscious group reported consuming food less frequently out of convenience (M = 4.7, SD = 1.3) or because it was quick to prepare (M = 4.8, SD = 1.2). Weight conscious also consumed foods less frequently because it was something they grew up with (M = 4.3, SD = 1.6) or because they felt like a particular food belonged to a certain situation (M = 3.4, SD = 1.4). Compared to breakfast skippers, weight conscious only rarely consumed food because it was trendy (M = 2.1, SD = 1.2). Taken together, breakfast skippers indicated a stronger social influence when compared to weight conscious given revealed preferences for foods based on occasions and family eating preferences.

## 5. Discussion

This study contributes to the literature in three main ways. This study sought to examine past segmentation studies to understand which segmentation bases have been used previously and confirms the importance of behavioural bases in segment formation and supports inclusion of demographic bases. Next, this study provides evidence of the value of including behavioural theory that extends focus beyond individual motivations for healthful eating. Finally, this paper demonstrates the application of a broader number of segmentation bases, together with behavioural theory, to derive segments in a young adult population. Each contribution is detailed in turn.

The importance of psychographic and behavioural variables in segment formation has been identified in previous research extending social marketing bases and using two-step cluster analysis [22,39]. Furthermore, the importance of psychographic variables has been demonstrated in segmentation analysis focusing on promotion of healthful eating habits [80]. Based on the predictor importance score, psychographic and demographic variables were the most important variables in segment formation in the current study. While demographic variables are not able to predict which people will perform a behaviour they will continue to play an important role in program planning and design given they can be directly linked to understanding where to locate programs and services and which communication channels to use. The results of this study support the continued inclusion of demographic variables on the grounds of the importance of this base in the current study.

The need to expand the behavioural theory base has been outlined [81]. This study demonstrated the ability of the MOA framework to explain eating behaviour, ensuring that understanding extends beyond psychographic factors (e.g., motivation) to consider the role the food environment exerts on an individual’s healthy eating. This study further establishes the utility of applying theory within segment formation. Past theoretically informed segmentation studies had applied individual focused theories, namely the Theory of Planned Behaviour [39] and the Theory of Interpersonal Behaviour [38]. These studies demonstrate how individual focused theories can deliver segments to inform program planning revealing differences in psychological predispositions between segments. This study advances understanding, applying the MOA framework, demonstrating the value of including a theory that takes environmental support into account. Past research has demonstrated superiority in variance explained for ecological models [82,83] when compared to individual Theory of Planned Behaviour models. Less environmental support was reported by breakfast skippers who also reported lower eating scores. These findings indicate that programs will need to focus on delivering targeted healthful eating solutions combined with communication messages in order to change motivations and deliver improvements.

These findings appear to be consistent with the predictions of the MOA framework when taken together with the average healthful eating behaviour of young adults in the segments [31]. It seems the more or less motivated each segment is to eat healthfully the more or less healthful eating is reported. Strategies that provide more healthful eating opportunities, for example, could possibly raise the motivation levels of young adults. Although empirically testing this relationship is beyond the scope of this study, future research could test this proposition within this context.

The study offers insights that can be used to change eating behaviour of young adults. Two distinct segments (breakfast skippers and weight conscious) were identified in the young adult population, each with unique beliefs about what motivates them to eat more healthfully, the different opportunities they have to eat healthily and their ability to make healthy food for themselves. For social marketers working on improving eating habits of young adults, this study delivers managerially useful insights. The two segments identified could be targeted by one or more social marketing interventions. Weight conscious are more motivated to eat healthily in order to maintain a balanced diet, because the food they eat is healthy and because they are conscious of their weight when compared to breakfast skippers. Weight conscious were more skillful and reported higher ability to purchase their own food and they reported higher availability of fruit and vegetables when compared to breakfast skippers. Weight conscious were more prone to eat vegetables and drink at least 1 litre of water on a daily basis when compared to breakfast skippers. Based on the results, social marketing interventions could target the attitudes of breakfast skippers to encourage positive perceptions of buying and cooking healthy foods to increase healthful eating behaviour. It could be beneficial for interventions to concentrate on reinforcing the beliefs of breakfast skippers that eating healthily is enjoyable and beneficial, especially since both their mean MOA and Turconi eating behaviour scores were lower when compared to weight conscious.

In addition to individual approaches outlined previously, social marketing interventions could enhance environmental support for healthful eating. Evidence demonstrating the value of these approaches is available. Consider, Carins et al. [84] who altered the food environment delivering an environment where paying customers chose more of the healthiest foods from the presented array of options (which had made healthier alternatives more prominent). While Sanigorski et al. [85] created healthy lunch (combo) packs to sell in school cafeterias during their program. Social marketers can encourage healthful eating behaviour of breakfast skippers by identifying mitigating factors and making healthful eating and breakfast consumption easier and more fun. Increasing the availability of healthful eating opportunities on the university campus, such as ‘come and try free healthy breakfasts’ followed by discount vouchers to food outlets on campus for both breakfast and lunch, would increase opportunities for healthful eating. Furthermore, increasing the number of healthy food outlets on campus should be a priority for university management. Social marketers may also negotiate with universities and local supermarkets to offer a student discount on fruit and vegetables through online delivery providers.

A recent analysis of Australian Health Surveys demonstrated that young adults (both men and women) from low socioeconomic backgrounds are at greatest risk of overweight and obesity [86]. With 95% of youth in Australia owning a smartphone, technology offers a prominent opportunity to reach and provide effective and sustainable interventions [87]. Online interventions (i.e., use of internet, mobile, or wireless devices to deliver services and information to improve health outcomes) have been announced as the way forward due to its pervasiveness and unique features when compared with traditional approaches [88]. Different and large parts of the population become accessible with online interventions and the flexibility of online platforms enables the adaption to segments ensuring tailored program delivery and simplification of complex concepts via video, graphics, and audio systems. Online interventions are also simple and low cost offering a significant advantage when compared to face to face interventions [89]. Social marketers may create an app or a website with segment specific information about easy to prepare healthy meals and recipe tips on how to cook a healthy meal. The intervention could include supporting features such as a food diary or a goal setting activity where breakfast skippers can set themselves goals to help reach healthier eating habits. These goals can include both a larger target goal (e.g., developing healthful eating habits) and smaller mid goals (e.g., drinking a glass of water every morning or eating a piece of fruit every day) to support the behaviour change process. Also, a video about how to identify healthy options and what to buy from a supermarket could be added as a part of the online intervention.

### Limitations and Future Research

This study is restricted by certain limitations. First, the study used a convenience, cross-sectional sample from several universities in one state of Australia and is therefore limited to a young adult university student sample within one metropolitan area in Australia. Therefore, any generalisations beyond the current sample are challenging. Future studies should look beyond the sample of this study and collect data from young adults in general beyond university settings, from every state and from people living in rural areas to ensure the segments are representative of the wider young adult Australian population. Future research utilising a longitudinal research strategy to investigate the long-term effects of healthful eating (e.g., reduction of unhealthy food consumption and increase in breakfast and fruit and vegetable intake) at a later period is recommended. Further research would help to understand if the segments are comprehensive, replicable and usable over time.

Furthermore, self-reported measures of behaviour were used. In social sciences self-report measures are the most widely used methods [90] however, self-reporting has its weaknesses due to social desirability bias and possible incorrectness of data caused by selective memory bias and these need to be taken into account [91]. Future research measuring actual eating behaviour through mechanical observations [92] could verify the correctness of self-reported data. Preferably, observations would be connected to individual self-reports.

A lack of previous research studies on the application of MOA in social marketing healthful eating studies [27] is acknowledged. Even though constructs were validated it is recommended to cross-validate the constructs on another population to extend understanding of the feasibility of applying MOA to change behaviour in the healthful eating context.

The current study was underpinned by three segmentation bases: demographic, psychographic, and behavioural. It is recommended to obtain a large enough sample size in order to include the geographic base, allowing all four segmentation bases to be analysed simultaneously. There has been no previous research seeking to understand whether some segmentation bases are more predictive of future behaviour than others. To examine the possible predictive effectiveness of segmentation bases a longitudinal experimental design is recommended. Such a study would test if segments formed on one base alone (e.g., demographics) are more predictive of future behaviour than segments drawn based on all four segmentation bases.

Although the application of segmentation is recommended, and evidence indicating that different segments respond to the same program differently [22,23], there is lack of evidence to determine if application of segmentation improves program effectiveness. A field experiment is recommended to investigate if a segmentation driven program that offers different options to different segments based on their wants and needs is more effective than a program where the entire target audience is treated the same way.

## 6. Conclusions

This study aimed to further examine the feasibility of market segmentation within the context of improving the eating habits of young adult university students. The results revealed two distinct segments within a healthful eating context that were defined by MOA framework constructs. Respondents within these segments had significantly different eating behaviours, motivations to eat healthily, abilities, and opportunities to purchase and make healthy foods, as well as different demographic profiles. The generalisability of the study is limited, and a broader study is recommended in order to extend understanding. Future research is required to examine if the identified segments respond to a social marketing interventions differently. This study provides evidence of the usefulness and feasibility of theoretically informed segmentation in social marketing aiming to change eating behaviour of young adults, an essential focus considering the growing frequency and consequence of overweight and obesity among young adults.

## Figures and Tables

**Table 1:** Social marketing segmentation studies encouraging healthful eating (2000–2018).

Author	Target Audience	Theory	No. of Segmentation Bases	Demographic	Geographic	Psychographic	Behavioural
Bryant et al. [42]	Women, infants, young children	**X**	2	✓	✓		
Casado & Rundle-Thiele [43]	Caregivers of school children	Exchange Theory	1			✓	
Chrysochou et al. [44]	Adults	Framework of discourses regarding consumer’s healthy eating	2	✓		✓	
Kazbare et al. [45]	13–15-year-old adolescents	Theory of planned behaviour	2	✓			✓
Keihner et al. [46]	Children	Resilience theory, social-cognitive theory	2	✓	✓		
Levine et al. [47]	Children	Social learning theory (SLT)	1	✓			
Naughton et al. [48]	Adults	Social cognition models	2			✓	✓
Neiger & Thackeray [49]	Adults	Stages of change theory (SCT)	1	✓			
Rosi et al. [50]	Children	**X**	1	✓			
Van Loo et al. [51]	Adults	Elaboration likelihood model	1			✓	
Young et al. [52]	Children	**X**	1	✓			

✓ = Segmentation base used; X= No mention of theory.

**Table 2:** Segment profile.

	Total 100% *n* = 327	Breakfast Skippers 48.6% *n* = 159	Weight Conscious 51.4% *n* = 168	*p*
Age *				**0.000**
20–24		77.7%	30.9%	
25–29		14.9%	40%	
30–35		7.4%	29.1%	
Education *				**0.000**
High school		100%	0%	
Graduate certificate		0%	7.1%	
Diploma		0%	16.7%	
Advanced diploma		0%	6.0%	
Bachelor’s degree		0%	38.7%	
Postgraduate degree		0%	24.4%	
**Motivation**		4.4 (1.4)	4.9 (1.2)	**0.002**
I eat what I eat				
to maintain a balanced diet *		4.7 (1.9)	5.2 (1.7)	**0.014**
because it’s healthy *		4.9 (1.4)	5.3 (1.4)	**0.026**
because I watch my weight *		3.7 (1.8)	4.2 (1.7)	**0.013**
**Ability**		4.5 (1.5)	4.9 (1.5)	**0.024**
because I have the skills to shop for my own food *		5.0 (1.8)	5.4 (1.7)	**0.023**
because I can make many different things		4.3 (1.9)	4.6 (1.8)	0.154
because I can cook many different things		4.3 (1.8)	4.6 (1.9)	0.062
**Turconi eating behaviour score**		15.9 (3.6)	16.9 (3.1)	**0.009**
You eat breakfast *		2.1 (1.0)	2.3 (0.9)	**0.044**
You eat at least 2 portions (200g) of fruit		1.7 (0.9)	1.7 (0.9)	0.762
You eat at least (200g) of vegetables *		2.1 (0.8)	2.3 (0.8)	**0.023**
You eat a cake or a dessert at meals		2.1 (0.7)	2.1 (0.6)	0.348
You drink wine or beer at meals		2.4 (0.7)	2.4 (0.7)	0.989
You eat 3 meals		2.1 (0.9)	2.2 (0.8)	0.186
You drink at least one glass of milk or you eat at least one cup of yoghurt		1.4 (1.1)	1.4 (1.1)	0.702
You drink at least 1–1.5L of water *		2.1 (1.0)	2.4 (0.7)	**0.002**
**Opportunity**		4.3 (1.5)	4.7 (1.5)	**0.041**
I eat what I eat				
because there are lots of different fruit and vegetables available *		4.4 (1.8)	4.9 (1.7)	**0.016**
because there are many shops selling fruit and vegetables nearby		4.0 (1.8)	4.3 (1.9)	0.225
because fruit and vegetables are easy to buy		4.5 (1.8)	4.8 (1.9)	0.163

^*^ Significant at the 0.05 level or less (bold).
